# Anti-malarial IgG subclasses pattern and FcγRIIa (CD32) polymorphism among pregnancy-associated malaria in semi-immune Saudi women

**DOI:** 10.1186/1475-2875-12-110

**Published:** 2013-03-21

**Authors:** Amre Nasr, Osama Hamid, Abdelhamid Al-Ghamdi, Gamal Allam

**Affiliations:** 1 Department of Microbiology, College of Medicine, Taif University, PO Box 888, Taif, Saudi Arabia; 2 Department of Microbiology, Faculty of Science and Technology, Al-Neelain University, Khartoum, Sudan; 3 Department of Basic Medical Sciences, College of Medicine, King Saud bin Abdulaziz University for Health Sciences, Riyadh, Saudi Arabia; 4 Department of Public Health, Jazan Health Affairs District MoH, Jazan, Saudi Arabia; 5 Department of Surgery, College of Medicine, Taif University, Taif, Saudi Arabia; 6 Department of Zoology, Faculty of Science, Beni-Suef University, Beni-Suef, Egypt

**Keywords:** Pregnant, Asymptomatic, Malaria, IgG, Subclasses, FcγRIIa, Polymorphism, Saudi Arabia

## Abstract

**Background:**

Pregnant women remain are at an increased risk of malaria with primigravidae being at the highest risk. Genetic polymorphism of the Fc receptor IIa for immunologlobulin (Ig) G (FcγRIIa) determines IgG subclass binding. Protection against pregnancy-associated malaria (PAM) is associated with the production of IgG specific for apical membrane antigen-1 (AMA-1). The present study was undertaken to examine the relationship between specific IgG/IgG subclasses and malaria infection. The second aim of the study is to examine the association between FcγRIIa R/H131 polymorphism in correlation with specific anti-malarial IgG antibodies of AMA-1 distribution and asymptomatic malaria infection among Saudi women living in the southern part of Saudi Arabia.

**Methods:**

One hundred and twenty pregnant women living in an area of meso-endemic *Plasmodium falciparum* malaria infection were consecutively enrolled onto the study. These pregnant women were asymptomatic and attending routine antenatal clinics. The levels of plasma antibodies (IgG and subclasses AMA-1) were measured using indirect enzyme-linked immunosorbent assays (ELISA). Genotyping of FcγRIIa-R/H131 dimorphism was performed using gene-specific polymerase chain reaction (PCR) amplification with allele-specific restriction enzyme digestion (*Bst*U1) of the PCR product.

**Results:**

A total of sixty-two (52%) pregnant women was diagnosed with asymptomatic malarial infection (ASM) compared with 58 (48%) malaria free controls (MFC). In the ASM group, there were high levels of anti-malarial IgG1 and IgG3, when compared to MFC (*P* value <0.001, respectively). The FcγRIIa-R/R131 genotype and R131 were found to be statistically significantly more prevalent in the ASM group when compared to the MFC group [55% for ASM *versus* 12% for MFC, odds ratio (OR) 5.62, 95% confidence interval (CI)= (2.03- 15.58), *P* value= 0.001]. However, the H/H131 genotype showed statistically significant association with MFC [14% for ASM *versus* 50% for MFC, OR(0.36), 95% CI= (0.14- 0.95), *P* value= 0.03].

**Conclusions:**

The study revealed that the ASM patients had higher anti-malarial IgG and IgG subclasses antibody levels when compared to the MFC. The FcγRIIa-R/R131 genotype and R131 allele were found to be statistically prevalent in the ASM when compared to the MFC group. The individuals carrying H/H131 were consistently associated with higher levels of anti-malarial IgG subclasses.

## Background

Malaria remains a devastating global health problem. The Arabian Peninsula lies at the fringes of malaria endemicity. Control efforts have successfully brought local transmission to a halt in many parts, with the exception of southern Saudi Arabia. In areas of intense parasite transmission, *Plasmodium falciparum* malaria is largely a childhood disease due to the gradual achievement of protective immunity. Each year, more than 125 million pregnant women are at risk of malaria infection with devastating consequences for the newborn and first and second time mothers [1]. In highly endemic settings, up to 50% of low birth weight (LBW) deliveries can be attributed to malaria in pregnancy. Pregnancy-associated malaria (PAM), which is characterized by accumulation of infected red blood cells (iRBCs) in the placental intervillous space [2-4], is a major cause of LBW and maternal anaemia in areas of endemic malaria transmission [5-7]. The pathogenesis of PAM and its association with adverse pregnancy outcome [8,9], such as intrauterine growth restriction and LBW is not well understood. One hypothesis for the adverse pregnancy outcome is the impairment in nutrient transport to the foetus [10], with possible effects on growth regulating hormones [11], and trophoblast invasion [12]. Previous *in vivo* studies showed the absence of adhesiveness to other receptor molecules mediating adhesion to receptors located only in placental tissue [2]. This may explain the susceptibility to malaria in otherwise clinically immune women at the time of their first pregnancy [4].

Antibody-dependent cellular mechanisms are thought to be central in the elimination of the parasite [13-16], and severe malaria is associated with increased pro-inflammatory immune response [17]. Immunoglobulin G1 (IgG1) and IgG3 are considered cytophilic and protective against *P. falciparum*. While low levels of IgG4 was thought to be relatively associated with protective mechanisms [15]. FcγRIIa exhibits a genetic polymorphism, FcγRIIa-R/H131 which codes for different capacities of IgG binding and phagocytosis [18-22]. Furthermore, IgG2, which binds the H131 allelic form of FcγRIIa, may be involved in human resistance to malarial infection [15,18,20]. A defence mechanism requiring the association of antigen bound IgG antibodies with FcγRs on monocytes is thought to play an important role in protecting immune African adults from malaria [18-20,23,24]. Only a few studies on FcγRIIa- R/H 131 polymorphisms in relation to malaria have been performed. A study in Western Kenya showed that the FcγRIIa 131H/H genotype was associated with enhanced susceptibility to placental malaria in HIV-positive, but not in HIV-negative women [25].

Hypothetically, immune responses against malaria can be distinguished into long- and short-term responses. Long-term response being broad and represented by pre-existing immunity, which may or may not be protective [26,27]. In contrast, the short-term immunity follows acute infection. Antibodies measured during acute infection or convalescence represent both boosted pre-existing and short-term immunity. However, it remains an open question as to whether a low level of malaria transmission is maintained throughout the dry season when clinical disease is absent. Furthermore, little is known about the relationship to the seasonal patterns of malaria transmission.

The present study was undertaken to examine the relationship between specific IgG/IgG subclasses and malaria infection. The second aim of the study is to examine the association between FcγRIIa R/H131 polymorphism in correlation with specific anti-malarial IgG antibodies of AMA-1 distribution and asymptomatic malaria infection among Saudi women living in the southern of Saudi Arabia.

## Methods

### Study area

This study was conducted in Jazan city in the southern Kingdom of Saudi Arabia (KSA), during the dry season, March to August 2012 (estimated population, 157,536 according to the 2010 census). The annual rainfall (between June and September) fluctuates from one year to another, and for that reason the incidence of malaria is markedly unstable. The uniqueness of this epidemiological setting is due to marked seasonality of low to moderate transmission of malaria. Whereas more than 90% of annual malaria incidence occurs between October and January; it is rare for a patient to develop more than one to two episodes of malaria during the season. Thus, it is easier to trace the immunological consequences of a single infection without interference of a superimposed infection. However, few sporadic cases of malaria may occur throughout the year.

### Study design and patient enrolment

A prospective cohort (cross-sectional) study was conducted in pregnant women attending routine antenatal clinics in King Fahad Specialist Hospital in Jazan (KFSHJ), without a clinical diagnosis of malaria. Patients with asymptomatic malaria are those without any of the classical symptoms of malaria infection as described by the WHO [28]. Patients were recruited after written informed consent was gained from them. Blood samples were taken for confirmation of malaria. Once patients were found to be positive for malaria, treatment was offered. Patients with asymptomatic malaria in their first trimester of pregnancy received quinine (20 mg/kg loading dose) plus clindamycine for seven days. Patients in their second and third trimesters received standard anti-malarial combination therapy (ACT). Standard ACT constitutes artesunate (4 mg/kg/day) given on days 0–2 and a single dose of sulphadoxine-pyrimethamine (25 mg/kg) given on day 0 [29-31]. All patients were reviewed by the study gynaecologist to confirm that there were no other co-infections apart from malaria. No one from the study groups was found to be HIV positive. All patients with other infectious diseases were excluded from the study. To control for the ethnicity factor, all the patients were from a Saudi background. The Saudi populations are originally Arab tribes living in the Arabian Peninsula for more than 1,434 years.

The study was reviewed and approved by the Ethics Review Committee in the College of Medicine at Taif University.

### Sample collection

Before pharmacological treatment was started, 50 μL of blood were collected on filter paper (Schleicher & Schuell; n° 903TM) for DNA amplification, parasite detection and measurement of immunoglobulin. One ml of venous blood sample was collected into EDTA Vacutainer® tubes (Becton Dickinson, Meylan, France) for parasite measurements. This was put into thick and thin blood smears obtained at the time of collection, which were then stained with Giemsa.

### Serum and DNA extraction

Serum extraction was eluted from a 50 μl blood drop onto filter-paper samples, as described previously [32,33]. DNA was extracted from a 50 μl blood drop onto filter paper with QIAamp® DNA Mini Kit (Qiagen, Hamburg, Germany), and re-suspended in 150 μl of TEB buffer [20].

### Parasite genotype

Five μl of DNA samples were used to detect the *P. falciparum* malaria parasite using a polymerase chain reaction (PCR), targeting for *ama-1_3D7* gene as described previously [34,35]. For the negative controls, we used blood samples collected from Swedish individuals who were never exposed to *P. falciparum* malaria parasites. For the positive controls, we used blood samples collected from Sudanese individuals who have been exposed to *P. falciparum* malaria parasites in the past.

### Enzyme-linked immunosorbent assays (ELISA)

IgG antibody was measured against the recombinant protein apical membrane antigen-1 (AMA-1), which has an N-terminal hexa-His tag, that is expressed in *Escherichia coli* and refolded *in vitro*. The expression and purification of these proteins was described earlier in [36,37]. The levels of serum antibodies (IgG total and subclasses) to malaria antigen (AMA-1) was measured using enzyme-linked immunosorbent assays (ELISA) as previously described [19,38]. EIA/RIA plates (Costar, MA, USA) were coated with AMA-1 at 1 μg/mL. The plates were incubated overnight at 4°C and then blocked for two hours with 0.5% bovine serum albumin (BSA) diluted in carbonate-bicarbonate buffer (pH 9.6). Plasma samples were diluted in incubation buffers (PBS + 0.5% BSA), 1:1000 for IgG and 1:400 for IgG subclasses (IgG1-4). Each sample was added in duplicates in the ELISA plate and incubated for one hour at 37°C. The plates were washed four times, and bound IgG antibodies were detected with goat anti-human IgG-ALP (1:2,000) (Mabtech, Nacka, Sweden). IgG subclasses were analysed with their respective biotin conjugated mouse anti-human subclass specific monoclonal antibodies: mouse anti-human IgG1 1:1,000 (M15015, Clone NL16, SkyBio, Bedfordshire, UK), mouse anti-human IgG2 1:3,000 (555874, Pharmingen, Erembodegem, Belgium), mouse anti-human IgG3 1:1,000 (MH 1532, Caltag Laboratories, Paisley, UK) and mouse anti-human IgG4 1:2,000 (B3648, Sigma, St. Louis, USA). Alkaline phosphatase (ALP) conjugated streptavidin (Mabtech) diluted 1:2,000 was added to detect bound antibodies of IgG2-4, while ALP-conjugated to goat anti-mouse Ig (Dakopatts, Glostrup, Denmark; 1:1,000) was used for IgG1 antibodies, and colour was developed with nitrophenyl phosphate (Sigma-Aldrich Chemie GmbH, Steinheim, Germany). The absorbance was read at 405 nm using a Vmax^TM^ Kinetic microplate reader (Molecular Devices, Menlo Park, USA).

### Statistical analysis

Statistical analysis was performed by SPSS version 10.0 software for Windows (SPSS®, Inc, Chicago, IL, USA). In this study, the antibody (IgG and IgG subclass) levels were analysed using Kruskal-Wallis test (non parametric) and the *P* values were derived. With regards to the risk of malaria infection during the pregnancy period, *P* values were considered significant if < 0.05. Overall, the 95% confidence intervals (CI) for odds ratio (OR) that did not cross 1.00 were statistically significance. OR above 1 represents values associated with the asymptomatic malaria group while less than 1 represents the malaria-free control group. To assess the association between the FcγRIIa genotype and malaria infection (dependent variable), logistic regression analysis was preformed with age and pregnancy duration as confounding factors. The FcγRIIa- Arg/ His 131 group was used as a reference in the analyses, as this is the genotype most prevalent in the human population [39]. The comparison of allele frequency was performed using a 2 × 2 chi-square test. To assess the relationship between the IgG (AMA-1) isotype levels and prevalence of parasite density in asymptomatic malaria patients; we used logistic regression analysis test.

## Results

### Characteristics of the study participants

A total of 120 pregnant women, were consecutively enrolled in the study and were classified into three groups according to pregnancy duration. Group I: First trimester [n=47 (39.2%); median age of 21 with a range of (18–26) years] (Table 1). Group II: Second trimester [n=33 (27.5%); median age of 22 with a range of (18–26) years] (Table 1). Group III: Third trimester [n=40 (33.3%); median age of 23 with a range of (18–26) years] (Table 1). Overall, there was statistical difference in the median and range of mother's age in the pregnancy duration *P* value= 0.046 (Table 1). A total of 58 women (48%); median age of 22 with a range of 18–26 years, were malaria parasite negative and had no clinical symptoms of malaria infection [malaria-free controls (MFC)]. A total of 62 women (52%); median age of 22 with a range of 18–26 years were malaria parasite positive and had no clinical symptoms of malaria infection [asymptomatic malaria (ASM)]. In the ASM patients, the median parasite density was 1,200 with a range of 250–2,000 parasite/μL). This is relatively low parasite density according to the WHO criteria [28]. There were no differences in age between the two study populations (median age 22 years for ASM *versus* MFC; OR= 1.29, 95% CI= 0.63–2.68, *P* value 0.486). All women attending the outpatient clinic at the time of enrolment were assessed initially by microscopy of blood film, and subsequent confirmation by PCR as mentioned in the parasite genotype section.

**Table 1:** Characteristics of the study groups, median (range) for (age, parasite density, IgG and IgG subclasses) regarding pregnancy duration

**Study groups variables**	**First trimester**	**Second trimester**	**Third trimester**	***P value***
**Malaria-free control n=58 (48%)**	13 (22.4%)	13 (22.4%)	32 (55.2%)	<0.001
**Asymptomatic Malaria n=62 (52%)**	34 (54.8%)	20 (32.3%)	8 (12.9%)	
**FcγRIIa genotypes**				
**R/R131 n=41 (%)**	22 (53.7%)	17 (41.5%)	2 (4.9%)	**<0.001**
**R/H131 n=41 (%)**	20 (48.8%)	11 (26.8%)	10 (24.4%)	
**H/H131 n=38 (%)**	5 (13.2%)	5 (13.2%)	28 (73.7%)	
**FcγRIIa alleles**				
**R 131 allele**	**0.68**	**0.68**	0.18	**0.004**
**H 131 allele**	0.32	0.32	**0.82**	
**Median (range) for:**				
**Age/years**	21 (18–26)	22 (18–26)	23 (18–26)	**0.046**
**Parasite density/μl**	1600 (250–2000)	1400 (510–2000)	1050 (250–2000)	**0.007**
**Specific antibody for AMA-1**				
**IgG**	1.28 (0.28- 6.04)	1.28 (0.50- 7.30)	2.76 (0.29- 6.90)	**0.003**
**IgG1**	0.59 (0.11- 21–95)	5.30 (0.16- 22.27)	5.43 (0.05- 32.89)	0.180
**IgG2**	0.04 (0.02- 5.64)	2.11 (0.02- 5.73)	2.16 (0.02- 6.10)	**0.040**
**IgG3**	0.52 (0.01- 37.94)	8.33 (0.12- 39.45)	8.39 (0.09- 46.00)	0.185
**IgG4**	0.02 (0.01- 2.62)	0.59 (0.01- 3.24)	0.61 (0.01- 2.01)	0.514

### Relative risk to malaria infection with parasite density, IgG and IgG subclasses in association with pregnancy duration

The characteristics of the pregnant women are shown in Table 1. The prevalence of malaria infection during the pregnancy duration was statistically significant, overall *P* value= <0.001 (Table 1). The pregnant women in the first and second trimesters showed higher incidence of malaria infection when compared with the pregnant women in their third trimester, *P* value <0.001. With regards to the prevalence of malaria infection in the first and second trimester, there was no statistical difference observed between the two groups *P* value= 0.253. There was statistically significant difference in the median parasite density of women in the third trimester compared to the first and second, *P* value= 0.007 (Table 1).

Overall, there were statistical differences with FcγRIIa genotype frequencies and pregnancy duration; *P* value <0.001 (Table 1). Furthermore; the frequency of R131 allele was higher within the first and second trimesters compared to women in the third trimester (Table 1). However, the H131 allele was of a significantly higher frequency in pregnant women during the third trimester compared to women in the first or second trimesters (Table 1). Overall, there was statistically significant difference between the allele frequency and pregnancy duration within the study groups; *P* value 0.004 (Table 1).

The median level of IgG and IgG2 were statistically different between groups within the pregnancy duration; *P* value= 0.003 and 0.040 respectively (Table 1). However, no statistical differences were observed between the median level of IgG1, IgG3 and IgG4 and the three trimesters (Table 1). In addition, the median levels of anti-malarial IgG and IgG subclasses were higher in the third trimester than the first trimester (Table 1).

### Patterns of antibody isotype in relation to relative risk of malaria infection *Plasmodium falciparum*-specific IgG and IgG subclasses

For anti-malarial IgG1 and IgG3, the ASM group showed very high levels compared to the MFC (*P* value <0.001, respectively) (Table 2). For the anti-malarial IgG antibody and IgG subclasses, the ASM showed significantly higher levels of IgG, IgG2 and IgG4 (*P* value 0.001, respectively) (Table 2). The most dominant subclass amongst the anti-malarial antibodies was IgG3, followed by IgG1, IgG2 and IgG4 in both study groups.

**Table 2:** Comparison between specific antibody (AMA-1) in asymptomatic malaria patients and malaria free controls

**Specific Ig antibodies**	**Median (Range) of the anti-malarial (AMA-1) antibodies**		***P value***
	**(MFC) n= 58 (48%)**	**(ASM) n= 62 (52%)**	
IgG	0.90 (0.28- 1.90)	3.36 (0.80- 7.30)	**0.001**
IgG1	0.20 (0.05- 1.58)	6.73 (4.90- 32.89)	**<0.001**
IgG2	0.41 (0.02- 0.16)	2.67 (1.84- 6.10)	**0.001**
IgG3	0.48 (0.01- 0.65)	11.32 (7.52- 46.00)	**<0.001**
IgG4	0.01 (0.01- 0.56)	0.75 (0.01- 3.24)	**0.001**

### Correlation of IgG subclass levels in relative risk of *Plasmodium falciparum* parasite density

The sixty-two patients diagnosed with ASM were investigated to determine whether the anti-AMA-1 IgG levels were associated with *P. falciparum* parasite density. Interestingly, there was statistically significant association between higher levels of IgG, cytophilic IgG1 and IgG3 subclass antibodies and a reduction in parasite density (lower than1,000 parasites/μL). The OR for IgG = 0.13; 95% CI: (0.02- 0.71) and *P* value = 0.005. The OR for IgG1 = 0.13; 95% CI: (0.03- 0.59) and *P* value = 0.008]. The OR for IgG3 = 0.5; 95% CI: (0.14- 1.74) and *P* value = 0.034] (Table 3). No significant associations were observed for non-cytophilic IgG subclasses and the parasite density (IgG2 and IgG4) (Table 3).

**Table 3:** Logistic regression analysis of the IgG (AMA-1) isotypes levels and prevalence of parasite density in asymptomatic malaria patients

	**Parasite density**	**Unadjusted OR; (95% CI)**	***P *value**
**IgG**	Higher than 1500 parasite/μl	11.61; (2.12- 13.73)	**0.018**
	1000- 1500 parasite/μl	1	
	Lower than 1000 parasite/μl	0.13; (0.02-0.71)	**0.005**
**IgG1**	Higher than 1500 parasite/μl	1.68; (0.25-11.23)	0.595
	1000- 1500 parasite/μl	1	
	Lower than 1000 parasite/μl	0.13; (0.03- 0.59)	**0.008**
**IgG2**	Higher than 1500 parasite/μl	2.00; (0.41- 9.78)	0.370
	1000- 1500 parasite/μl	1	
	Lower than 1000 parasite/μl	0.54; (0.14- 2.07)	0.392
**IgG3**	Higher than 1500 parasite/μl	6.33; (1.15- 15.01)	0.275
	1000- 1500 parasite/μl	1	
	Lower than 1000 parasite/μl	0.500; (0.14- 1.74)	**0.034**
**IgG4**	Higher than 1500 parasite/μl	3.69; (0.82- 6.66)	0.371
	1000- 1500 parasite/μl	1	
	Lower than 1000 parasite/μl	1.85; (0.48- 7.06)	0.089

### FcγRIIa-R/H 131 genotype and allele frequency in relation to asymptomatic malaria

All subjects within the two study groups were genotyped for the G494A single nucleotide polymorphism (R131H) in the FcγRIIa gene (rs1801274). Both allele and genotype frequencies were found to be in Hardy-Weinberg equilibrium (*P* value= 0.813) (Table 4). R/R131 genotype was dominant amongst ASM patients compared to MFC (55% for R/R *versus* 31% for R/H genotypes; unadjusted OR= 5.62; 95% CI (2.03-15.58), *P* value= 0.001 and adjusted OR= 5.11; 95% CI (1.74- 15.00), *P* value= 0.003) (Tables 4 and 5). The homozygote H/H131 genotypes was found to be statistically significant at higher frequency in MFC individuals compared to ASM patients [50% for H/H *versus* 40% for R/H genotypes; unadjusted OR= 0.36; 95% CI (0.14- 0.95), *P* value = 0.03] (Tables 4 and 5). Following the adjustment of the OR for pregnancy duration and age of pregnant women, there appears to be no statistical significance [adjusted OR= 0.75; 95% CI (0.26- 2.62), *P* value= 0.75] (Tables 4 and 5). With regard to FcγRIIa allele frequencies, there were significant differences between the ASM and MFC individuals, the R131 allele being more frequent than the H131 allele amongst ASM patients (0.70 *versus* 0.30), OR 3.31, 95% CI (1.59–6.56), *P* <0.001 (Table 4).

**Table 4:** Distribution of the FcγRIIa-131 and allele frequency in relation to asymptomatic malaria compared with malaria free control

**FcγRIIa genotypes**	**Malaria-free control n=58 (48%)**	**Asymptomatic malaria n=62 (52%)**	***Hardy-Weinberg equilibrium test***
**R/R131 n=41**	7 (12%)	34 (55%)	0.813
**R/H131 n=41**	22 (40%)	19 (31%)	
**H/H131 n=38**	29 (50%)	9 (14%)	
Alleles frequency*			
**R 131 allele**	0.31	**0.70**	
**H 131 allele**	0.69	**0.30**	

^*^ OR = 3.31, 95% CI (1.59–6.56); *P* value <0.001.

**Table 5:** Logistic regression analysis of the FcγRIIa-131 in relation to asymptomatic malaria compared with malaria free control

**FcγRIIa genotypes**	**Unadjusted OR; (95% CI) and *P *value**	Adjusted^* ^**OR; (95% CI) and *P *value**
**R/R 131**	5.62; (2.03- 15.58), **0.001**	5.11; (1.74- 15.00), **0.003**
**H/R 131**	1	1
**H/H 131**	0.36; (0.14- 0.95), **0.03**	0.75; (0.26- 2.62), 0.75

* Adjustment of OR was performed for pregnancy duration and age.

### Analysis of anti-malarial antibodies in relation to FcγRIIa genotype

The distribution of FcγRIIa genotype and IgG subclass profile in the subject groups were analysed. *Kruskal-Wallis* (nonparametric) test showed that IgG3 antibodies specific to AMA-1 malarial antigen were associated with individuals carrying the R/R131 genotype than H/H131 genotype in the ASM group (*P* value <0.001) (Table 6). High levels of anti-malarial IgG1, IgG2 and IgG4 antibodies specific to AMA-1 malarial antigen, were associated with individuals carrying the R/R131 genotype than the H/H131 genotype in the ASM group (*P* value =0.021, 0.021 and 0.006 respectively) (Table 6).

**Table 6:** Correlations between the median values of IgG (AMA-1) antibodies responses and distribution of FcγRIIa 131 genotypes in risk of asymptomatic malaria

	**FcγRIIa 131 genotypes**	**IgG**	**IgG1**	**IgG2**	**IgG3**	**IgG4**
		Median (Range)	Median (Range)	Median (Range)	Median (Range)	Median (Range)
**Malaria free controls**	**R/R**	0.60 (0.50- 0.80)	0.62 (0.16- 1.41)	0.10 (0.02- 0.13)	0.15 (0.12- 0.62)	0.02 (0.02- 0.03)
	**R/H**	0.95 (0.28- 1.90)	0.17 (0.10- 1.55)	0.04 (0.02- 0.13)	0.46 (0.01- 0.65)	0.01 (0.01- 0.56)
	**H/H**	1.10 (0.29- 1.80)	0.17 (0.05- 1.58)	0.04 (0.02- 0.16)	0.50 (0.01- 0.65)	0.01 (0.01- 0.56)
**Asymptomatic malaria**	**R/R**	3.43 (0.80- 7.30)	7.12 (4.90- 32.89)	2.79 (1.84- 6.10)	12.50 (7.52- 46.00)	0.86 (0.01- 3.24)
	**R/H**	3.75 (0.90- 6.42)	6.67 (4.95- 21.22)	2.62 (1.98- 5.60)	10.65 (7.97- 37.58)	0.73 (0.59- 1.88)
	**H/H**	2.67 (0.80- 5.64)	5.48 (4.91- 11.51)	2.21 (1.95- 3.64)	8.40 (7.78- 20.99)	0.59 (0.01- 1.70)
	*P *value*	0.661	**0.021**	**0.021**	**<0.001**	**0.006**

^*^P value was derived from *Kruskal-Wallis* test (nonparametric).

## Discussion

Despite the importance of malaria in pregnancy, this may be the first report on the immunology of *P. falciparum* in pregnant Saudi Women. This report investigated the relationship between specific IgG, IgG subclasses (AMA-1) antibodies, and FcγRIIa R/H131 polymorphism in association with asymptomatic malaria infection among Saudi women living in southern KSA. The risk of maternal infection was highest during the first and second trimester. One possible explanation may be that CD4^+^ and CD25^+^ regulatory T cells, which increase during the third trimester, modulate the immune response [40]. The levels of CD4^+^ and CD25^+^ regulatory T cells gradually returns to normal level after pregnancy, thus reflecting the pattern of increased incidence of malaria during the first and second trimesters [40]. Specific Th1–type immune responses are pivotal for successful immunity to malaria. These are altered during pregnancy, which might diminish the ability to control new infections and asymptomatic parasitaemia. As a consequence, pregnant women are put at an increased risk of the development of malaria [41].

When assessing the relationship between specific IgG, IgG subclasses (AMA-1) antibodies and pregnancy duration, it appears that there is a significant difference in IgG and IgG2 between the study groups and the duration of pregnancy. Furthermore, the levels of anti-malarial IgG and IgG subclasses were higher in the third trimester than the second and first trimesters. This finding may offer an explanation to the lower prevalence of malaria infection in the third trimester when compared to the first and second. In support of this finding, a previous study in Mali by Dicko *et al.* reported the first trimester as the main risk period for malaria infection [42]. Furthermore, a recent study in Sudan showed that pregnant women in their second trimester were more susceptible to malaria infection [43].

The current study showed accumulative association of high levels of IgG and IgG subclass antibodies with ASM patients. In support of this finding, a previous cohort study in Gabon showed that pregnant women who had malaria infection had higher levels of specific IgG of variant surface antigens (VSA) antibody compared to women who had no malaria infection [44].

The correlation of anti-malarial IgG, IgG subclass antibodies and malaria infection showed that the serum levels of IgG1 and IgG3 subclass antibodies were found to be higher than IgG2 and IgG4 antibodies, for *P. falciparum* blood-stage AMA-1 antigen. The cytophilic antibodies (IgG1 and IgG3) are currently thought to be instrumental in protective immunity, while non-cytophilic antibodies against the same epitopes may block the protective activity of cytophilic ones [16,23,45]. Several sero-epidemiological studies have supported these findings by indicating an association between protection from malarial infection with antibodies of IgG1 and IgG3 subclasses [20,45,46]. Furthermore, when comparing the antibody levels between the ASM and malaria free controls, the ASM showed significantly higher levels of IgG2 and IgG4 antibodies reactive to AMA-1. Previous studies from different endemic areas in Africa, showed a correlation between the severity of malarial infection and high circulating levels of IgG2 [15,20,47].

When assessing the relationship between anti-malarial IgG, IgG subclass antibodies and the risk of parasite density, there were higher levels of anti-malaria AMA-1 antibody IgG, IgG1 and IgG3. This may have played a crucial role in parasite reduction. The suggested mechanism by which these subclasses are protective involves their binding to the FcRs on monocytes, leading to antibody dependent cell mediated inhibition of parasite replication [23,45]. This is supported by the results from this study, since the two most predominant IgG subclasses, are IgG1 followed by IgG3. All of the above mechanisms may have affected the parasite density and, therefore, leading to infected patients without clinical symptoms.

The importance of FcγRIIa with regards to malaria comes from its role as a link between the humoral and cellular branches of the immune system [48]. In the present study, the FcγRIIa genotype was related to PAM in Saudi women. FcγRIIa-R/R131 and R131 genotype were associated with ASM compared to MFC. However, FcγRIIa-H/H131 genotype and H131 allele had lower frequency in ASM compared to MFC. A previous study in western Kenya suggested that FcγRIIa- H/H131 genotype was associated with enhanced susceptibility to placental malaria in HIV positive women but not in HIV negative women [25]. Studies in different settings suggested that the FcγRIIa-R/R131 genotype protects against high levels of parasitaemia, whereas the FcγRIIa-H/H131 genotype was associated with susceptibility to severe malaria with high parasite burden [49-51]. An additional study in Sudan showed an association between FcγRIIa-R/R131 and severe malaria [20].

The present study was undertaken to examine the relationship between polymorphism in the gene for primary cellular IgG receptor, FcγRIIa and PAM. This study has provided evidence that the high levels of IgG subclasses are associated with FcγRIIa-R/R131 genotype among ASM women in southern Saudi. The results are in contrast with a study in western Kenya which described evidence that the IgG2-binding FcγRIIa-H/H131 genotype is associated with increased susceptibility to placental malaria in HIV-positive women [25].

Although, the FcγRIIa are important molecules in malaria outcome; several studies have attempted to associate mutations in these genes with increased susceptibility to developing different forms of malaria. However, the results are contradictory and no conclusion has yet been determined. This confliction may be due to differences in the degrees of malarial endemicity, and genetic background of the individual as well as the differences in the study designs.

## Conclusions

Taken together, this study revealed that the ASM patients showed higher anti-malarial IgG and IgG subclass antibody levels when compared to the MFC. Furthermore, FcγRIIa-R/R131 genotype and R131 allele were associated with ASM patients. However, the FcγRIIa-H/H131 genotype is associated with higher levels of anti-malarial IgG subclass. Thus, the higher IgG subclass antibody levels seen in the ASM may be due to the relatively high prevalence of the individuals carrying the H/H131 genotype in this study group. Expectedly, the result of the present study suggests that the magnitude and the quality of anti-malarial humoral response is not the only key element for relative reduction in malaria infection in the ASM group. Further studies are needed to elucidate if the FcγRIIa-R/H131 polymorphism is a contributing factor to the differential susceptibility to malaria seen in different groups.

## Competing interests

The authors declare that they have no competing interests.

## Authors’ contributions

OH and GA designed the study and carried out the sampling; AN and GA performed the ELISA; AN performed the genotyping of FcγRIIa polymorphism and participated in the statistical analysis. Both AN and GA drafted the manuscript. AN, AAG and GA set up the framework, financed and revised the manuscript. All authors participated in the manuscript preparation, read and approved the final version of the manuscript.
